# Study of Collagen Birefringence in Different Grades of Oral Squamous Cell Carcinoma Using Picrosirius Red and Polarized Light Microscopy

**DOI:** 10.1155/2015/802980

**Published:** 2015-10-26

**Authors:** Pillai Arun Gopinathan, Ganganna Kokila, Mahadesh Jyothi, Chatterjee Ananjan, Linganna Pradeep, Salroo Humaira Nazir

**Affiliations:** ^1^Oral & Maxillofacial Pathology, Sri Sankara Dental College, Varkala, Akathumuri, Vennicode, Kerala 695318, India; ^2^Oral & Maxillofacial Pathology, Sri Siddhartha Dental College & Hospital, Sri Siddhartha Academy of Higher Education, Tumkur 572107, India; ^3^Oral & Maxillofacial Pathology, Vananchal Dental College & Hospital, Farathiya, Garhwa, Jharkhand 822114, India

## Abstract

*Objectives*. The present study was done to evaluate birefringence pattern of collagen fibres in different grades of oral squamous cell carcinoma using Picrosirius red stain and polarization microscopy and to determine if there is a change in collagen fibres between different grades of oral squamous cell carcinoma. *Materials and Methods*. Picrosirius red stained 5 *μ*m thick sections of previously diagnosed different grades of squamous cell carcinoma and normal oral mucosa were studied under polarization microscopy for arrangement as well as birefringence of collagen fibres around tumour islands. *Results*. It was found that thin collagen fibres increased and thick collagen fibres decreased with dedifferentiation of OSCC (*P* < 0.0001). It was observed that there was change in polarization colours of thick fibres from yellowish orange to greenish yellow with dedifferentiation of OSCC indicating loosely packed fibres (*P* < 0.0001). *Conclusion*. There was a gradual change of birefringence of collagen from yellowish orange to greenish yellow from well to poorly differentiated squamous cell carcinoma, indicating that there is a change from mature form of collagen to immature form as tumour progresses. Studying collagen fibres with Picrosirius red for stromal changes around tumour islands along with routine staining may help in predicting the prognosis of tumour.

## 1. Introduction

Squamous cell carcinoma (SCC) is the commonest type of malignancy affecting oropharyngeal region [1]. SCC is primarily composed of malignant epithelial cells and stroma in which they are dispersed [2]. The birefringence of collagen is related to its physical aggregation which could be altered due to action of collagenases, matrix metalloproteinases (MMPs) secreted by tumour cells [2–4]. In present study, an attempt was made to observe if there is any change in nature of collagen fibres in different grades of oral SCC (OSCC) by determining the ratio of thick to thin fibers as well as their polarizing colours in Picrosirius red stained sections.

## 2. Materials and Methods

Formalin fixed, paraffin embedded tissue blocks of 50 diagnosed cases of OSSC were retrieved from archives of Department of Oral and Maxillofacial Pathology, Sri Siddhartha Dental College, Tumkur. Of these 20 cases were well differentiated SCC (WDSCC), 20 cases were moderately differentiated SCC (MDSCC), and 10 cases were poorly differentiated SCC (PDSCC). 10 cases of clinically uninflamed, normal oral mucosa (NM) were obtained during minor oral surgical procedures. The protocol for the research has been approved by Institutional Ethical Committee.

Two 5 *μ*m thick sections were prepared from each paraffin embedded tissue block using semiautomatic microtome. One of these sections was stained with hematoxylin and eosin and the other with Picrosirius red stain. Hematoxylin and eosin stained sections were viewed under bright field microscopy and the cases were segregated as well, moderately, and poorly differentiated OSCCs according to WHO grading system based on degree of keratinization, cellular and nuclear pleomorphism, and mitotic activity [5, 6].

Areas showing epithelial ulceration and dense inflammatory cell infiltration in connective tissue were excluded as inflammation is said to have an impact on packing of collagen fibres. In the normal tissues collagen fibres from lamina propria were studied, while in OSCCS collagen fibres around tumour islands were studied. The polarization colours were determined for 50 thin fibres (0.8 *μ*m or less) and 50 thick fibres (1.6–2.4 *μ*m) in each tissue sample. To evaluate change in ratio of thick and thin collagen fibres in different grades of OSCC, randomly 50 fibres were observed and segregated into thick and thin fibres.

### 2.1. Statistical Analysis

To eliminate subjective bias two observers autonomously evaluated all cases. The obtained scores were tabulated and subjected to statistical analysis using one-way analysis of variance test (ANOVA) for intragroup significance.

## 3. Results

**Figure 1:**
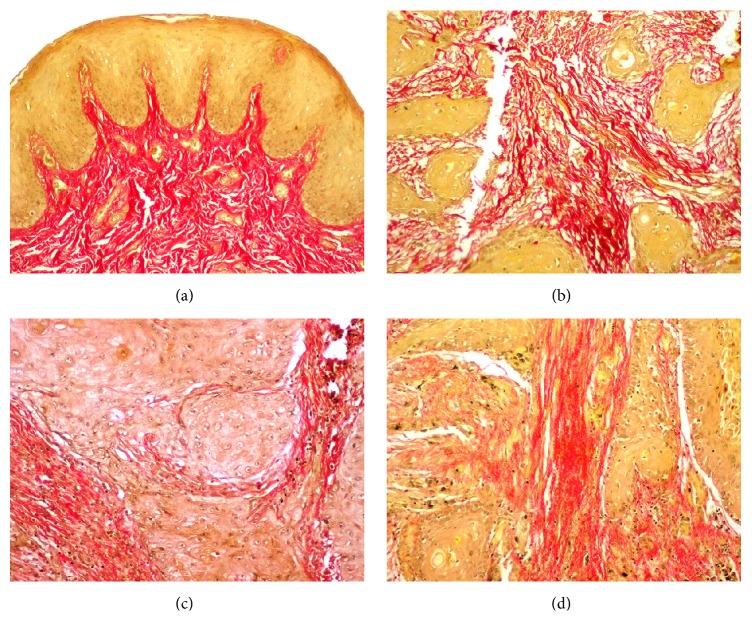
Photomicrograph of PSR stained sections under bright field microscope (10x) showing collagen stained deep red. (a) NM. (b) WDSCC. (c) MDSCC. (d) PDSCC.

**Figure 2:**
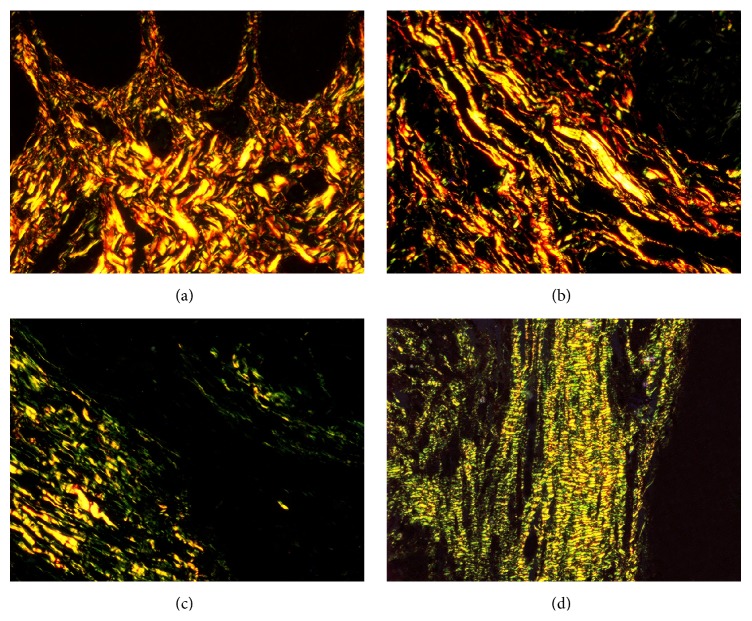
Photomicrograph of PSR stained sections under polarized light microscopy (20x). (a) NM showing YO birefringence. (b) WDSCC showing predominantly YO birefringence. (c) MDSCC showing YO to GY birefringence. (d) PDSCC showing predominantly GY birefringence.

**Figure 3:**
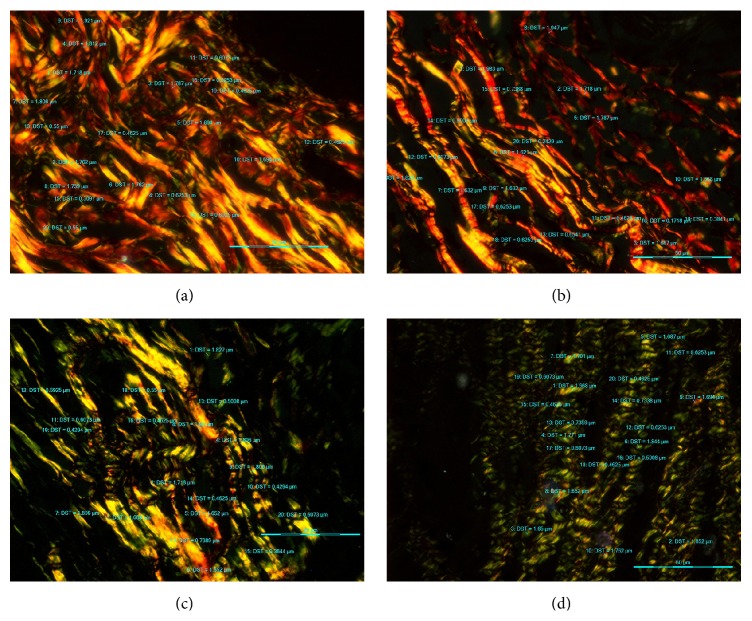
Photomicrograph of PSR stained sections under polarized light microscopy (40x). (a) NM showing predominantly thick YO collagen fibres. (b) WDSCC showing predominantly thick YO and thin GY collagen fibres. (c) MDSCC showing thick YO and thick GY collagen fibres. (d) PDSCC showing thick GY and thin GY collagen fibres.

The Picrosirius red stained sections of NM, WDSCC, MDSCC, and PDSCC under bright field microscopy showed collagen fibers stained deep red (Figures 1(a), 1(b), 1(c), and 1(d)). On examining under polarization microscopy at a lower magnification NM and WDSCC predominantly showed YO birefringence (Figures 2(a) and 2(b)). In MDSCC both YO and GY birefringence was observed (Figure 2(c)), whereas in PDSCC GY birefringence was predominantly seen (Figure 2(d)). At a higher magnification in NM and WDSCC thick collagen fibres with YO birefringence were seen (Figures 3(a) and 3(b)). In MDSCC thick collagen fibres showed YO as well as GY birefringence (Figure 3(c)), whereas in PDSCC thick collagen fibres were predominantly GY (Figure 3(d)).

### 3.1. Collagen Fibre Arrangement in the Connective Tissue Stroma of NM, WDSCC, MDSCC, and PDSCC

**Figure 4:**
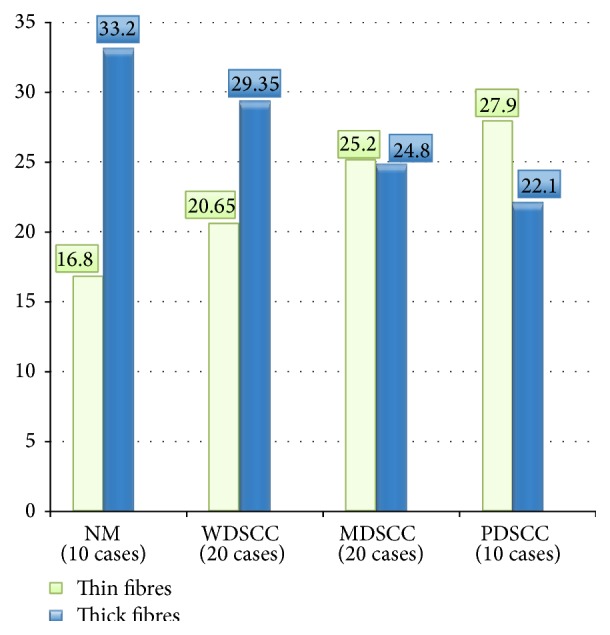
Thin and thick fibres arrangement in NM, WDSCC, MDSCC, and PDSCC (out of 50 fibres). ANOVA analysis: *F* value: 124.31; *P* value: 0.0000; interpretation: highly significant.

The Picrosirius red stained sections of NM, WDSCC, MDSCC, and PDSCC were studied under polarized light microscopy for type of collagen fibre arrangement in the connective tissue. It was observed that thin fibres increased with dedifferentiation of OSCC which was statistically significant (*P* < 0.0001) (Figure 4). The thick fibres decreased with dedifferentiation of SCC which was also statistically significant (*P* < 0.0001) (Figure 4).

### 3.2. Polarization Colours of Collagen Fibres in the Connective Tissue Stroma of NM, WDSCC, MDSCC, and PDSCC

**Figure 5:**
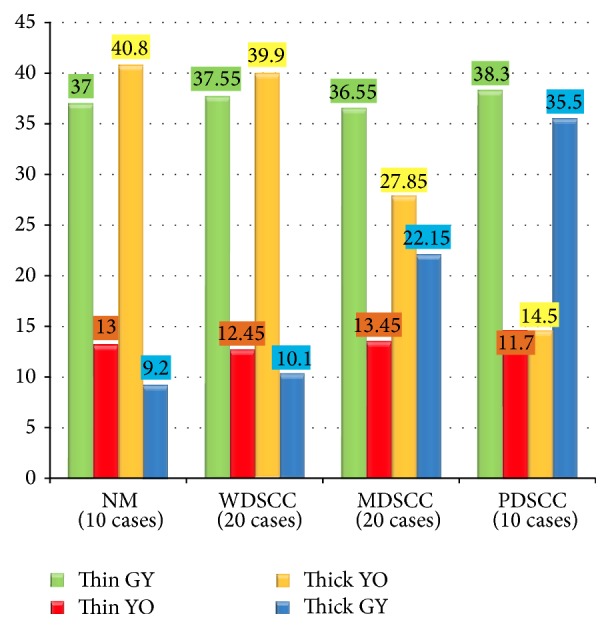
Polarization colours of thin and thick fibres in NM, WDSCC, MDSCC, and PDSCC cases. ANOVA analysis: for thin fibres: *F* value: 1.607; *P* value: 0.1979; interpretation: not significant. For thick fibres: *F* value: 224.90; *P* value: 0.0000; interpretation: highly significant.

Examination of the stroma of NM, WDSCC, MDSCC, and PDSCC in PSR stained sections showed that polarization colours of thin collagen fibres were predominantly greenish yellow (Figure 5). While polarization colours of thick collagen fibres were mainly yellowish orange in NM and WDSCC, they gradually changed to greenish yellow in MDSCC and PDSCC mainly around tumour islands (Figure 5).

The changes in polarization colours of thin collagen fibres in the stroma of NM, WDSCC, MDSCC, and PDSCC were not statistically significant (*P* value: 0.1979) (Figure 5). But the changes in polarization colours of thick collagen fibres in the stroma of NM, WDSCC, MDSCC, and PDSCC were statistically significant (*P* < 0.0001) (Figure 5).

## 4. Discussion

OSCC is a common malignancy in India, accounting for 50–70% of total cancer mortality [7]. Carcinomas are composed of diverse cell populations that are heterogenous for a wide range of characteristics. The tumour progression is accompanied by degradation of the basement membrane and components of matrix which occurs at several stages of metastatic cascade, including local invasion, angiogenesis, and vascular and lymphatic invasion [8].

The mechanical quality of ECM is mainly dependent on its collagenous content and it is the presence of collagen which is considered a main barrier to be cleared away during invasion, thus making room for infiltrating cell mass [9]. MMPs are a group of proteolytic enzymes which degrade most of the components of ECM. The MMP system consists of 23 MMPs which are further divided into five groups, namely, gelatinase, collagenase, stromelysins, membrane type MMPs, and less well characterized MMPs [8, 10]. The extracellular matrix mainly consists of type I collagen which is about 90% and type III collagen which is 8–10% [11]. Electron microscopic studies have shown that type I collagen fibres are coarse and are composed of closely packed thick fibrils, whereas type III collagen forms thin fibres and are composed of loosely disposed thin fibrils [12].

In the present study there were statistically significant (*P* < 0.0001) increase in number of thin collagen fibres and decrease in number of thick collagen fibres which was evident by observing the collagen fibre arrangement in different grades of OSCC. The increase in thin fibres and decrease in thick fibres with dedifferentiation of OSCC could be due to the initial fibroproliferative response and in later stages there will be abnormal collagen production and defective maturation which may promote the neoplastic growth [13]. In extracellular matrix of skin tumour there was an increase in deposition of type I and type III collagen fibres in the stroma of WDSCC, but the destruction of fibrillary structures was more pronounced during the decrease of differentiation from MDSCC to PDSCC [14]. Studies on respiratory neoplasm have also shown that total collagen volume decreases with increasing degree of malignancy from WDSCC, MDSCC to PDSCC and also collagen fibre size decreased in less differentiated SCC [15].

In a study on maturation of type I and type III collagen fibers in different grades of endometrial adenocarcinoma it was found that in well differentiated adenocarcinomas a distinct layering of type I collagen and bundles of stromal fibres formed a solid homogenous stroma between epithelial cell arrangements. In moderately differentiated adenocarcinomas fibres were irregular and there was a weak deposition of type I collagen adjacent to tumour islets, whereas poorly differentiated adenocarcinomas showed a sparse stroma surrounding individual tumour cells and increased deposition of type III collagen [13].

Lysis of stroma is an essential requirement for invasive growth. It is seen that collagen disintegrates that is they undergo “elastotic degeneration”. Electron micrographic studies have shown diffuse collagenolysis and phagocytosis of intact collagen fibrils in the course of carcinoma. Malignant epithelial cells produce various lytic enzymes like cathepsin, elastolytic and glycosaminoglycan degrading enzymes which attack the stroma and induce the fibroblast to synthesize collagenolytic activities [9].

MMPs are a family of proteases and have a generic role in clearing ECM components from the path of a migrating tumour cell. Most of the epithelial tumours express MMPs which are found initially in the surrounding tumour stroma. MMPs exert their effects by proteolyzing the available substrates; for example, MMP-2 induces cell migration, MMPs 2, 3, and 7 release TGF*β*1, and Str-1 (MMP-3) causes cell apoptosis. Other collagenolytic enzymes implicated during tumour growth include lysosomal enzymes particularly acidic cathepsin which attacks collagen fibrils at nonhelical telopeptide regions [16].

A study on collagen in different histological stages of oral submucous fibrosis (OSMF) showed change of polarization colour of thick collagen fibers from YO to GY in advancing connective tissue stages and degrees of epithelial dysplasia [17]. OSMF is a cytokine and growth factor induced disease which increases collagen deposition with advancement of disease [18], while in SCC there is degradation of collagen fibres with advancement of the disease. Thus, it could be hypothesised that there are increase in thin collagen fibres and simultaneous decrease in thick collagen fibres with dedifferentiation of OSCC.

Numerous studies have been done on PSR polarization method and have been used in dental and gingival research to demonstrate pathological changes in collagen [19]. Collagen when stained with PSR and when viewed under polarized light microscopy normally shows thin collagen fibres (type III) which are green to greenish yellow, while thick collagen fibres (type I) range from yellowish orange to orange red polarization colours [3, 4, 12]. The green to greenish yellow colour of both thin and thick fibres suggests that the collagen is loosely packed and orange red colour originates from tightly packed fibres [3, 4]. The particular colours produced by polarization microscopy of PSR stained section could be due to fibre size, alignment and packing, cross-linking of fibres, interstitial ground substance, and water content. It is also seen that, in tightly packed and better aligned collagen molecules, a shift to the longer wavelength of polarization colours was seen [20, 21].

Polarization colours of collagen fibres in the fibrotic process have shown that, during maturation of fibres, the proteoglycan content changes and dehydration occurs which increases the number of cross-links and stainable side groups; thus the diameter of collagen fibres grows markedly. Finally all these factors enhance the intensity of birefringence and at the same time change their polarization colours. Thus young, very fine type I collagen fibres with weak birefringence appear green in colour similar to the mature type III fibres. They become orange or red in the further maturative stage [22].

Collagen is birefringent, which is used to assess collagen organization and microstructure. The degradation of collagen in pathologies results in disorganization and loss of polarization sensitivity [23, 24]. Optical technologies like polarized microscopy and Polarization-Sensitive Optical Coherence Tomography are used to assess tissue birefringence which is indicative of disease progression [24]. Polarized microscopy works by passing a natural light through a polarizer called as Nicol prism [25], while Polarization-Sensitive Optical Coherence Tomography works by assessing the polarization state of back-reflected light [24]. Further 2H Double Quantum Filtered (DQF) Nuclear Magnetic Resonance (NMR) spectrum studies have shown that green to greenish yellow colour of thin and poorly packed collagen fibres correlates the narrow component of 2H DQF NMR spectrum, while yellow orange red colour pattern of thick well packed collagen fibres corelates the broad component of spectrum [26]. In the present study with respect to relationship between collagenous components in the stroma adjacent to tumour cells, observable changes have been noticed in different histological grades of OSCC. The polarization colours of thin fibres were similar in all the study groups presenting mainly greenish yellow colours. The changes in polarization colours of thin fibres in the stroma of the different study groups were not significant (*P* value: 0.1979). The polarization colours of thick fibres showed a gradual change from predominantly yellowish orange (YO) to greenish yellow (GY) with dedifferentiation of OSCC. The changes in the polarization colours of thick fibres in stroma of different study groups were significant (*P* < 0.0001).

In the present study the polarization colours of thick fibres were YO 79.8% in WDSCC and 55.7% in MDSCC mainly around the tumour islands, which could be due to deposition of collagen fibres in the form of thick bands and closely packed fibrils [2]. The change in polarization colours of thick fibres in MDSCC and PDSCC showed a gradual change in birefringence from YO to GY around the tumour islands, which could be due to loosely packed fibres which might be composed of procollagens, intermediate or pathological collagen rather than normally tight packed fibres. The change in the birefringence of thick fibres in the present study could also be due to adjacent tumour cells which secrete enzymes such as collagenases or MMPs, disorganized stroma, and uninhibited proliferation of dedifferentiated tumour cells with secretion of their abnormal matrix [5]. A similar change in the polarization colours of collagen fibre ranging from YO to GY was observed in the different grades of OSCC carried out by Aparna and Charu [2].

The observations on capsular collagen staining of follicular thyroid carcinomas by PSR showed higher frequency of yellow green collagen fibres at the site of invasion and this could be due to decreased packing density or size of fibre. On the other hand, invasion may result in collagen degradation due to either proteolysis induced by tumour or mechanical pressure due to growth of tumour. It was also noted that orange red fibres were predominant in the noninvaded sites of the follicular thyroid carcinoma [21]. In a study on stromal difference in salivary gland tumour pleomorphic adenoma (PA), polymorphous low grade adenocarcinoma (PLGA), and adenoid cystic carcinoma (ACC) it was found that polarization colours of thin fibres in all the lesions were predominantly greenish yellow, whereas polarization colours of thick fibres in PLGA and ACC were equally divided between greenish yellow and yellow orange and predominance of yellowish orange fibres was seen in PA [15]. A study on human osteosarcoma revealed the presence of type III collagen fibres in anaplastic areas, while both types I and III collagen were present in the fibroblastic areas of the tumour [27].

Green to greenish yellow polarization colours of thick fibres were also noted in pathological conditions like central odontogenic fibroma [28], ameloblastic fibroma [29], odontogenic keratocyst [3], advanced cases of OSMF [18], oxodipine induced hyperplastic gingivitis [30], anastomotic site of intestine [31], and connective tissue nevi [32]. This change in the polarization colours of thick fibres from yellow orange to greenish yellow is considered due to loosely packed fibres which might be composed of procollagens, intermediate or pathological collagen rather than normal tight packed fibres [3, 4, 28–32].

In the present study, an obvious stromal change with the dedifferentiation of the neoplasm was clear with PSR. There were a significant increase of thin and decrease of thick collagen fibres with the dedifferentiation of OSCC. When the birefringence of collagen fibres in the OSCC cases was observed, in well differentiated squamous cell carcinoma, there was deposition of collagen in the form of thick bands revealing thick yellowish orange fibres adjacent to the neoplastic epithelial islands. Gradually in moderately and poorly differentiated squamous cell carcinoma there was a change in the polarization colours of thick fibres from yellowish orange to greenish yellow, where the fibres were fibrillar and more disorganized. This definitively indicates the contribution of the stromal constituents in the progression of the neoplasm which could aid in predicting the prognosis of the tumour.

## 5. Conclusion

On the basis of the present study it may be concluded that with dedifferentiation of OSCC there was a change in the polarization colours of thick fibres from YO to GY due to abnormal collagen production, degradation, and defective maturation which could promote neoplastic progression. In this study, change in the ratio of type I and type III collagen distribution along with change in the birefringence of thick collagen fibres specifically to the degree of dedifferentiation of the neoplasm was seen.

## Abbreviations

NM: Normal mucosa
WDSCC: Well differentiated squamous cell carcinoma
MDSCC: Moderately differentiated squamous cell carcinoma
PDSCC: Poorly differentiated squamous cell carcinoma
YO: Yellowish orange
GY: Greenish yellow
PSR: Picrosirius red.
